# When should Home-visit nurses initiate end-of-life discussions for patients with Organ failure and family caregivers? A qualitative study

**DOI:** 10.1186/s12912-023-01401-x

**Published:** 2023-08-07

**Authors:** Kurumi Asaumi, Masataka Oki, Yoshie Murakami

**Affiliations:** 1 Department of Nursing, School of Health Sciences, Tokyo University of Technology, 5-23-22 Nishikamata, Ota-ku, Tokyo, 144-8535 Japan; 2 Faculty of Nursing, Toho University, 4-16-20 Omorinishi, Ota-ku, Tokyo, 143-0015 Japan

**Keywords:** End-of-life discussions, Home-visit nurses, Patients with organ failure, Timely identification, Qualitative descriptive study, Japan

## Abstract

**Background:**

End-of-life (EOL) discussions for organ-failure patients with family caregivers are important factors for successful EOL care. However, identifying the appropriate time to initiate these discussions is difficult owing to the unpredictability of the disease trajectory. No practical tools or clinical indicators currently exist that can help identify non-cancer patients receiving home care who need EOL discussions.

**Methods:**

The survey was conducted from February 2020 to June 2021. To identify the appropriate time at which to initiate EOL discussions for patients with organ failure and their caregivers, we determined the time when home-visit nurses initiated EOL discussions. We interviewed 19 home-visit nurses (mean total home-visit nursing experience: 6.7 ± 5.9 years) and analyzed the data using Hsieh and Shannon’s qualitative content approach.

**Results:**

Three themes related to home-visit nurses’ experiences of identifying the appropriate time to start EOL discussions were identified: symptomatic worsening, lack of patients’ and family caregivers’ EOL awareness, and decline in activities of daily living.

**Conclusions:**

It is necessary to develop a tool that will enable home-visit nurses to implement EOL discussions at the appropriate time.

**Supplementary Information:**

The online version contains supplementary material available at 10.1186/s12912-023-01401-x.

## Background

Population aging rates are rapidly accelerating worldwide [1]. It is anticipated that Japan’s aging society will peak in 2040, with high mortality [2]. According to a Japanese government survey, 70.6% of citizens wished to die at home in case of severe heart failure [3]; however, 73.0% and 13.2% of all deaths in Japan occurred in hospitals and homes, respectively, in 2017 [4]. Previous systematic reviews indicated that end-of-life (EOL) care at home increases the possibility of death at home and slightly improves patient satisfaction without raising costs [5, 6]. In this context, there is an urgent need to create a community-based integrated care system so that people can live out their lives in their own, familiar communities. Home-visit nursing systems that offer EOL care based on patients’ wishes at home are considered a cornerstone of community-based integrated care [7].

Discussions regarding EOL care allow patients and caregivers to examine their goals and expectations for the medical care they want to receive as the patient’s death approaches [8, 9]. Thus, such discussions play a key role in successful EOL care. Previous studies have shown that high-quality discussions about EOL care improved the quality of the care provided and reduced the family’s depression [9–11].

Concerning the types of injury or disease that home-visit nursing agencies in Japan encounter, cardiovascular diseases accounted for the largest share (24.8%), followed by mental and behavioral disorders including dementia (16.1%), neurological diseases (15.8%), and malignant neoplasms (8.9%) [12]. However, the appropriate time to initiate EOL discussions cannot be uniformly determined for organ-failure diseases whose trajectory, unlike cancer, is hard to predict—such as heart failure or chronic obstructive pulmonary disease [13]. This is because patients with organ-failure diseases typically experience periods of deterioration and recovery over time [14]. Data have shown a marked difference between the proportion of heart failure patients (7%) on palliative care registers compared with cancer patients (48%); patients with heart failure are registered for palliative care much later than those with cancer [15]. Less than 10% of patients with chronic kidney disease reported having discussed EOL care in the past year [16], and a Japanese survey showed that non-cancer diseases are a risk factor for a lack of patient participation in EOL discussions [17]. Among the reasons reported by previous studies for this are healthcare providers’ fears, lack of communication skills, focus on curative therapies, and limited time and space to discuss EOL care [18]. Moreover, patients with advanced chronic obstructive pulmonary disease, chronic heart failure, and chronic renal failure sometimes change their preferences for where to die and preferences for life-sustaining treatment over the course of one year [19, 20]. Thus, discussions should be conducted when patients approach death to identify how and where they wish to spend their remaining life and should be repeated at key milestones in the disease trajectory of a patient with organ failure [21]. EOL discussions are usually conducted by physicians or specialists who can explain the disease and prognosis. However, in Japanese home-care settings, home-visit nurses who understand patients’ medical and lifestyle needs spend the most time with patients in home care [7]. Consequently, they play a crucial role in multidisciplinary collaborations with relevant health and social service providers regarding EOL discussions.

Discussions about overall life goals, future medical decisions, and real-time medical decision-making should be initiated at all ages and all stages of health [22]. That said, the timing of EOL discussions is important because patient preferences change over time, and decision-making about life-sustaining treatments is context dependent [23]. Initiating EOL discussions too early might not accurately reflect patients’ wishes over time; conversely, initiating EOL discussions too close to death may result in hasty decisions or patients being unable to make decisions [23]. One systematic review indicated that discussions about EOL care should occur at certain clinically meaningful times, such as when a patient’s condition deteriorates or there is a significant decline in physical function [24]. Furthermore, considering the influence of sociocultural beliefs in EOL, the emphasis on personal autonomy—a fundamental Western concept—might not be valued equally in EOL decision-making in all cultures [25], especially in high-context cultures that prefer euphemisms, such as Japan. Although indicators and tools have been developed to help identify patients in need of palliative care [26–28], no such indicators or tools exist for identifying non-cancer patients receiving home care who need EOL discussions that also account for the influence of sociocultural beliefs.

Relatively few qualitative studies of EOL discussions for patients with organ failure in home-care settings are present. Examples include studies of the ability of general practice nurses to facilitate conversations about advance care planning [29], barriers to facilitating advance care planning for professionals (e.g., a lack of time) [30], the lack of disease-specific communication skills, and uncertainty about timing [31]. This study, therefore, explored how home-visit nurses determined when EOL discussions are necessary for patients and families through semistructured interviews and qualitative content analysis, thereby helping nurses to identify patients and families in need of EOL care discussions and when to implement them in home-care settings. We believe this study will aid the development of tools or clinical indicators to implement EOL discussions with patients with organ failure at the appropriate time.

### Setting: home-visit nursing system in Japan [7]

In Japan, home-visit nursing can be accessed under health insurance or long-term care insurance depending on the patient’s condition. It is provided for patients at any stage of illness when their primary care physician recognizes that home-visit nursing is needed and issues an order for it. Such home care can include monitoring health conditions, guiding the client’s recovery, rehabilitation, support for family caregivers, assistance with daily living activities (e.g., bath or toilet assistance), medical management, pressure ulcer prevention, wound care, and EOL care. Aside from a registered nurse license, home-visit nursing does not require any special licenses or qualifications. These caregivers work in hospitals, clinics, and home-visit agencies.

### Research purpose

This study aimed to gain insight into EOL discussions to enable home-visit nurses in Japan to identify the appropriate time at which patients with organ failure and their families need these discussions. In this study, we defined EOL discussions as repeated discussions home-visit nurses have with patients and their family caregivers regarding specific medical treatments (e.g., cardiopulmonary resuscitation), individualized medical treatment plans (e.g., palliative care options), patients’ life goals and values (e.g., comfort, independence, dignity) [22], where and how they want to spend their remaining time, and the chosen place of death at the end of life.

## Methods

### Study design

We used a qualitative descriptive design, following Sandelowski [32]. We chose this method for two reasons: (i) to explore the events that led nurses to initiate EOL discussions based on their description of the situation, and (ii) to summarize the data using everyday language that facilitates the understanding of these situations [32, 33].

### Sample and recruitment

The survey was conducted from February 2020 to June 2021. We used snowball sampling to select 19 home-visit nurses in Japan. The first author informed the first home-visit nurse who was interviewed about the study’s requirements and asked them to refer other participants via email or telephone. Inclusion criteria for participants were (i) a minimum of six months’ home-visit nursing experience and (ii) experience with conducting EOL discussions for terminal patients with organ failure, excluding patients with severe dementia who could not communicate with others.

### Data collection

Data for analysis were collected through semistructured interviews with participants. A single interview was conducted with each participant, either online or face-to-face. Participants were interviewed at their convenience in quiet locations at their home-visit nursing agencies. The interviews lasted between 20 and 42 min. Participants were asked to freely describe their perceptions and experiences. Before the interview, we asked them to choose a patient with whom they had held EOL discussions. At the beginning of the interview, participants’ basic demographic information and patients’ baseline clinical information were obtained. The interview questions were determined after the researchers’ consultation based on the first author’s clinical experience as below: (i) When did you decide to initiate EOL discussions with terminally ill patients with organ failure and/or their family caregivers? (ii) How did you initiate EOL discussions with patients with organ failure and/or their family caregivers? (iii) Why did you implement EOL discussions at that time? (iv) What happened to your patients and/or their family caregivers before and after the EOL discussions? During the interviews, participants’ facial expressions, speech, and silences were noted through reflective notes, which aided in the analysis. All interviews were transcribed verbatim using a digital voice recorder. We collected data from 19 home-visit nurses because of the unlikelihood of additional data from other nurses changing the results [34]. A professional transcription company was hired to create the transcriptions and we signed a nondisclosure agreement with them.

### Data analysis

Data collection and analysis were conducted concurrently. We added participants’ facial expressions, speech, and silences noted during the interviews to the interview transcriptions before data analysis. We analyzed the data using conventional qualitative content analysis [35]. Coding was done as follows. Codes were generated and used to sort the interview data in a way that best summarized, integrated, and represented the content. We read the data thoroughly and repeatedly, immersing ourselves to understand the content as a whole. Subsequently, we coded the data according to chunks of meaning, grouping similar codes into 16 categories to gain insight into larger themes. The subcategories were analyzed again to identify similarities and differences and then grouped into seven subthemes. The final thematic scheme was presented as a tree diagram with data belonging to each category.

We analyzed the data in Japanese, which was translated into English for this study. The professional translator’s and editor’s corrections were reexamined by the authors to ensure an adequate English–Japanese fit.

Lincoln and Guba’s framework [36]—which is related to the credibility, transferability, dependability, and confirmability of results—was used to ensure rigor in this study. Data triangulation was employed to ascertain credibility by observing and noting participants’ facial expressions, speech, and silences during the interviews. We added various descriptions that explained background information about the patient, their family, and other details of the circumstances leading to EOL discussions to improve transferability. To promote dependability and confirmability, the researchers held several meetings to develop codes, categories, and themes after the first author had coded the data. Any disagreements were discussed until consensus was reached. The coding process was documented in an Excel file to track the analysis and provide an overview of the process.

### Ethical considerations

This study was approved by the Ethics Committee of Tokyo University of Technology (no. E19HS-019), which the first author is affiliated with. All participants provided written informed consent. They were informed that participation in the study was voluntary and that they could withdraw at any time, without any negative impact. Before the interview, participants were assured that the interview would be interrupted immediately if they felt distressed while recalling their experiences. Additionally, close attention was paid to participants’ mental changes before and after the interviews. To protect their identities, audio-recorded and transcribed interview data were anonymized by substituting individual names with numbers. Data were stored in password-protected files and stored in a locked laboratory.

## Results

### Participant characteristics (table 1)

We interviewed 19 home-visit nurses working at 12 home-visit nursing agencies (11 in Tokyo and 1 in Hiroshima). There were five administrators, four oncology-certified nurse specialists, two palliative clinical specialists, and one home-care clinical specialist. Their ages ranged from 20 to 50 years. The mean total years of nursing experience was 16.4 ± 8.4, and that of home-visit nursing experience was 6.7 ± 5.9.

**Table 1:** Participants’ characteristics (n = 19)

Characteristic		n
Age range	20s	3
	30s	7
	40s	5
	50s	4
Years of nursing experience	< 10	5
	10–19	7
	≥ 20	7
Years of home-care nursing experience	< 5	10
	5–9	3
	10–19	4
	≥ 20	2
Sex	Male	1
	Female	18
Administrator	Yes	5
Oncology clinical nurse specialist	Yes	4
Palliative clinical specialist	Yes	2
Home-care clinical specialist	Yes	1

### Characteristics of patients and family caregivers

We gathered information about participants’ patients from the home-visit nurses’ recollections to contextualize their experiences. The patients included 13 men and 6 women with ages ranging from 70 to 90 years. Twelve patients lived with their family caregivers. Regarding family caregiver demographics, seven were spouses, three were children, one was grandchild, and one was sister. Patients’ primary diagnoses varied: eight had cardiovascular disease, five had respiratory disease, four had cerebrovascular disease, and two suffered from renal failure. Most had multiple comorbidities, such as hypertension and diabetes, as they aged. The actual place of death reported by participants was home for seven patients.

**Table 2:** Themes and subthemes

Themes	Subthemes
Symptomatic worsening	Patient feels physical or mental distress. Patient is repeatedly hospitalized and discharged. Near-death period approaches.
Lack of patients’ and family caregivers’ EOL awareness	Nurses discuss patient’s wishes and views on life and death in daily conversation. The prognosis, as perceived by the healthcare provider, differs from the perception of patients/family caregivers.
Declining ADL	Basic ADL decline becomes significant. Readjustment of long-term care services and the care environment.

### Timely identification of need for eol discussions by home-visit nurses (Table 2)

Three themes were identified to describe participants’ experiences of identifying the optimal time to start EOL discussions: symptomatic worsening, lack of patients’ and family caregivers’ EOL awareness, and decline in activities of daily living (ADL).

#### Symptomatic worsening

Changes associated with the symptoms of the disease prompted participants to initiate EOL discussions. They picked up signs of physical and mental distress from patients’ subjective statements, repeated hospitalizations and discharges, and clinical data about approaching death. Details on these subthemes are shared below.

**When the patient feels physical or mental distress.** Although patients with cancer might begin to prepare for death at the time of diagnosis, patients with organ failure cannot imagine death at the time of diagnosis and do not spend much time thinking about death. Therefore, home-visit nurses often initiate the sensitive topic of EOL care when they perceive that the patient feels out of sorts. The following quotations from the interview data illustrate this subtheme:

During the visit, I [the home-visit nurse] sensed something “out of the ordinary” about the convalescent from the dark expression on the face of the patient [with arrhythmia and mild dementia] and asked, “Is there something on your mind?” Then, I recognized that he was feeling “unwell.” Then, in light of the fact that he had lost four kilograms in two to three weeks, I thought it was time to ask about his thoughts in depth. (Nurse #6)

The patient [with pneumonitis] complained, “I want to die now. But I cannot pass away quickly because I don’t have cancer.” I then asked, “Since death will come someday, how do you want to spend your life now?” I began with a discussion that focused on how to live in the present. (Nurse #3)

**When the patient is repeatedly hospitalized and discharged.** Patients with organ-related disorders such as heart, lung, liver, and kidney disease experience repeated acute exacerbations over time, with repeated hospitalizations and discharges. In the case of acute exacerbations, even when the treatment is successful, it is not uncommon for the patient’s functional level to decrease. Therefore, participants concluded that repeated hospitalizations and discharges were a predictor of imminent death and that EOL discussions were necessary, as is evidenced by the following quotations.

I focus my talk on “what to do when the condition next worsens” because it is difficult to predict the prognosis for heart failure, and the patient will get better and worse repeatedly. When the patient has pleural effusion and difficulty breathing, it is not possible to ask, “Where do you want to spend your remaining time?” Therefore, I talk to him when he is in a better state. (Nurse #13)

Three months before his death, after several hospitalizations and discharges with pneumothorax after idiopathic interstitial pneumonia, we started EOL discussions progressively. (Nurse #19)

After the second discharge from the hospital, the doctor and I discussed with the patient [with heart failure] how far she wanted to go with treatment. (Nurse #10)

**When the near-death period approaches.** The final stages of organ failure often involve a series of acute exacerbations, followed by relatively rapid deterioration and death. Therefore, such situations indicated that EOL discussions should be conducted to confirm family caregivers’ final intentions since death was predicted to be only a few days away, as illustrated in the quotation below.

Based on the symptoms of “inadequate oral intake, the need for suctioning due to poor swallowing condition, significant lupus and edema, and low blood pressure,” I predicted that the patient [with heart failure] would die within a couple of days and felt that we must confirm with families the place of death again. (Nurse #8)

As the patient’s daughter watched the gradual decline of the patient [with cerebral infarction and aspiration pneumonia], she said, “It’s coming soon [death],” and I realized that she could accept death, and I began to talk about the final end-of-life care. (Nurse #17)

#### Lack of patients’ and family caregivers’ EOL awareness

The average time from initiating home nursing care to death is 11.95 months for patients without cancer, compared with 3.32 months for patients with cancer in Japan [37]. In the case of patients without cancer, it is necessary to confirm not only the patient’s intention for home care but also the family caregiver’s preparedness and understanding of circumstances, considering the long period during which the family caregiver’s life will be affected. Details on these subthemes are shared below.

**When nurses discuss the patient’s wishes and views on life and death in daily conversation.** Home-visit nurses, who understand both patients’ medical and lifestyle needs, provide daily direct care at home, listen to patients’ feelings and wishes during their daily care, and initiate EOL care following patients’ wishes. The following quotations from the interview data illustrate this subtheme:

Through daily conversations and reminiscences, “what they value” and “perceptions about life and death” of patients [with heart failure], which is the center of EOL discussions, were captured. (Nurse #10)

Patients [with heart failure] are end-stage heart failure patients who live alone, so I began EOL discussions at the time of discharge when he confirmed his clear intention to live alone at home without much help from others. (Nurse #13)

**When the prognosis, as perceived by the healthcare provider, differs from the perception of patients/family caregivers.** Although it is difficult to predict with precision when death will occur in patients without cancer, healthcare providers make approximate prognoses based on their clinical experience and medical knowledge. However, there is often a discrepancy between their prognosis and the family’s perceived time of death owing to a lack of knowledge or family health issues such as dementia. Therefore, home-visit nurses believed it was necessary to understand the gap between their prognosis and the patient/family caregiver as a prerequisite for proceeding with EOL discussions, as illustrated in the quotations below.

The home-care team [with doctor, home-visit nurse, visiting caregiver, care manager] understood that treatment was ineffective and wanted to help the patient [with chronic obstructive pulmonary disease] achieve the “end-of-life care at home” that he wanted, but the family preferred “transportation in case of emergency” and felt the need to discuss the matter. (Nurse #2)

I suggested to the sister of the patient [with atrial fibrillation and stroke] that she call two other family members to discuss EOL care because the level of consciousness and vital signs were fluctuating. But I couldn’t communicate with her smoothly; I supposed the sister may have dementia. I believe that we have to include other family members in discussions about where to die and how to care for EOL. (Nurse #9)

The patient [with heart failure] was always at risk of worsening heart failure because he liked fatty foods and would eat and drink on his own, except for meals prepared by helpers. Therefore, it is necessary to have a discussion that includes the fact that worsening disease could lead to death. (Nurse #15)

#### Declining ADL

The need to readjust long-term care services and environments owing to changes associated with ADL prompted home-visit nurses to begin EOL discussions.

**When basic ADL decline becomes significant.** Home-visit nurses considered the timing of starting EOL discussions after grasping the changes in their basic ADL, such as getting up, transferring, moving, eating, changing, toileting, bathing, and dressing, as illustrated in the quotations below.

When that patient [with arrhythmia and mild dementia] fell or soiled himself by not making it to the bathroom in time, we asked, “How do you perceive the recent change in your activities of daily living?” The patient responded, “I’m feeling a little weak,” so I felt that this was an opportunity [for an EOL discussion]. (Nurse #6)

The patient’s [cerebrovascular disease] physical condition was stable for about six months after the home-care team intervened, but gradually the patient’s falls increased, even indoors, and her grandchildren became concerned about continued home care and talked about her future place of care. (Nurse #1)

The patient [with interstitial pneumonia] was unable to walk to the bathroom on his own and was unable to eat, triggered by the fever. At that time, his family members were present, and his mental state was stable; therefore, we turned the conversation toward how to spend his final time. (Nurse #2)

**When to readjust long-term care services and care environment.** As noted above, the average time from initiating home nursing care to death for patients without cancer is longer than that for cancer patients. Thus, participants assessed the family caregivers’ burden, recommended utilizing long-term care services to reduce the burden, and started to discuss EOL care. The following quotations from the interview data illustrate this subtheme:

I believe that the progression of the disease [old myocardial infarction] will increase the frequency of home-visit nursing, including emergency visits, which is also when EOL discussions should be initiated. (Nurse #7)

The patient [with cerebral hemorrhage and aspiration pneumonia] gradually experienced an increased loss of stools and ADL decline; thus, the burden on his wife increased. Thus, we talked about predictable patient changes from now until the patient passed away and recommended adjusting services, such as increasing the frequency of home-visit nursing and home care and using a short stay. (Nurse #16)

## Discussion

To provide quality EOL care, it is essential to initiate or conduct EOL discussions with patients and their family caregivers at the appropriate time. Three themes were identified to describe participants’ experiences of identifying the optimal time to start EOL discussions: symptomatic worsening, lack of patients’ and family caregivers’ EOL awareness, and ADL decline. This study is one of the few to examine home-visit nurses’ timing of EOL discussions for patients with organ failure and their family caregivers in Japan. In the absence of consensus in international clinical practice about when to initiate or conduct EOL discussions with patients with organ-failure diseases for whom the disease course is difficult to predict [13], our findings could provide guidance for decision-making about when to initiate EOL discussions (which are the foundation of EOL care).

First, participants initiated EOL discussions based on changes in the patient’s physical condition, and the timing was determined from subjective information such as “something is different than usual” that patients themselves observed or from objective information such as weight loss, vital signs, or repeated hospitalizations and discharges. Older adult patients without cancer often have multiple diseases associated with aging, which makes it difficult to assess symptomatic worsening. Therefore, patients’ awareness that there was “something wrong,” along with objective data, helped participants determine the starting point of EOL discussions. In addition, our findings correspond to those of another study that found that general practitioners initiated advance care planning for patients with organ failure after a period of exacerbation, such as a hospitalization [38]. According to a review [25], patients and caregivers had mixed feelings about timing, and many preferred to defer EOL discussions until they perceived them to be clinically relevant during periods of serious illness. Considering this, we believe an appropriate time to initiate EOL discussions is when patients feel out of or at a clinically relevant time in the illness trajectory (e.g., in case of exacerbation).

Second, a previous scoping review recommended that patients’ preferences in dealing with EOL challenges should be valued [39], while others recommended encouraging patients and family caregivers to share their concerns about the patient’s medical condition, clarify their goals and values for EOL care, and discuss their EOL care preferences [40, 41]. We believe these results are similar to our findings that home-visit nurses repeatedly discussed EOL care to bridge the knowledge gap, based on how patients and family caregivers understand the medical condition, values, and preferences that are identified through daily care. We believe this action promotes the process of EOL discussion. Moreover, cooperation between professionals from the medical, nursing, and welfare fields, as well as community-based medical care programs, should be considered, because the difficulties of handling EOL discussions with other healthcare providers were not brought up by the participants during the interviews. Other multidisciplinary EOL education intervention programs that promote seamless collaboration between medical and social service professionals have had a positive effect on inter-professional collaboration between home-visit nurses and welfare workers [42]. Considering this, we believed that home-visit nurses maintained a good relationship with doctors and other medical and welfare providers through discussions about readjusting long-term care services and the care environment.

Additionally, it is important to pay attention to the health issues of family caregivers who live at home for long periods. In the case of Nurse #9, the primary caregiver, the older sister, was suspected of having dementia, and the EOL discussion did not go smoothly. A scoping review noted that bad communication and relationships between patients, families, and healthcare providers led to suspicion or confusion about the circumstances surrounding the death [39]. Home-visit nurses should explore the physical and mental state of key family caregivers who are in charge of EOL discussions in case of difficulty in communication.

Third, it is important to consider that the Japanese have a high-context culture [43]. The National Institute for Health and Care Excellence guidelines state that “patients should be clearly informed” when the prognosis is predicted to be a few days [44]. However, reportedly 67% of hospice cancer patients in Taiwan and 20% in South Korea are told that their life expectancy is a few days, while in Japan only 5% are informed of this [45]. This is because, given the influence of sociocultural beliefs at EOL, the emphasis on personal autonomy—which is a fundamental Western concept—might not be equally valued in all cultures in the context of EOL decision-making [25]. Contrarily, another study reported that only 33% of family members of patients with cancer regretted not having talked about death sufficiently [46]. Further, families who made preparations for death—such as spending time with the patient, seeing people the patient wanted to see, and making funeral arrangements—were less likely to experience depression and complicated grief, regardless of whether they clearly talked about death [47]. Considering our findings and these results, discussions about readjusting long-term care services and care environments that are based on home-visit nurses foreseeing death might facilitate patients’ and families’ engagement in preparing for death without talking about death directly. However, family caregivers might experience distress, confusion, and uncertainty regarding what to expect if the patient’s poor condition is explained through euphemisms [39, 48]. Thus, we believe that healthcare providers must choose words and topics carefully when they intend to help to prepare for death without talking about death directly. Our results could be applicable to other high-context cultures.

### Implications for practice

Given that population aging is accelerating rapidly, and considering the social need for home-visit nurses in Japan, it has been estimated that by 2025, Japan will need 120,000 home-visit nurses [49]. In 2021, however, only 60,000 nurses were working at home-visit nursing agencies [50]. Therefore, with the current supply of home-visit nurses not matching the demand, we believe it is necessary to consider how to deliver EOL care efficiently and effectively. We propose that one solution is to assess the optimal timing to initiate EOL discussions with due regard for the characteristics of patients without cancer and family caregivers. In this regard, we believe our findings can contribute to the development of a tool for assessing the appropriate timing for such discussions. Assessing the optimal timing to start EOL discussions would enable home-visit nurses to help patients and families prepare for death without talking about death directly or causing depression.

In future research, it could be useful to collect big data nationwide across Japan to support a predictive model based on machine learning, allowing the determination of the most adequate moment to initiate an EOL discussion suited to each patient’s needs.

### Study Limitations

This study has some limitations. First, we asked home-visit nurses to recall earlier discussions with patients and family caregivers, increasing the possibility of recall bias. Second, we collected data in a very restricted perimeter, around the Tokyo metropolitan area; thus, our findings cannot be generalized to other prefectures of Japan with a more rural identity. Third, we collected only restricted demographic information; therefore, other factors such as educational level, socioeconomic status, and religion, to name a few, might also influence these attitudes toward EOL discussion initiation.

## Conclusion

In this study, we explored how home-visit nurses identified the appropriate time at which patients and families need to begin EOL discussions in Japan. Based on a qualitative content analysis of interview data from 19 home-visit nurses, three themes were identified to describe home-visit nurses’ experiences of identifying the optimal time to start EOL discussions: symptomatic worsening, lack of patients’ and family caregivers’ EOL awareness, and declining ADL. It is necessary to develop a tool that will enable home-visit nurses to implement EOL discussions for patients and family caregivers at the appropriate time.

### Electronic supplementary material

Below is the link to the electronic supplementary material.

Supplementary Material 1

## Data Availability

The datasets generated and/or analyzed in this study are not publicly available because the data contain individual participant information, but they are available from the corresponding author upon reasonable request.

## Abbreviations

EOL: End-of-life
ADL: Activities of daily living
