# Supplementary material for: When should Home-visit nurses initiate end-of-life discussions for patients with Organ failure and family caregivers? A qualitative study
Source: BMC Nurs. 2023 Aug 7;22:258. doi: 10.1186/s12912-023-01401-x (PMC10405459; doi:10.1186/s12912-023-01401-x)
Supplement: Supplementary file 1 — Supplementary Material 1 [file 12912_2023_1401_MOESM1_ESM.docx]

|  | **Appendix 1. *Interview guide*** |
| --- | --- |
| 1 | When did you decide to initiate and implement EOL discussions for terminally ill patients with organ failure and/or their family caregivers? |
| 2 | How did you initiate and implement EOL discussions for terminally ill patients with organ failure and/or their family caregivers? |
| 3 | Why did you implement EOL discussions at that time? |
| 4 | What happened to your patients and/or their family caregivers before and after EOL discussions? |
